# Knowledge mapping and bibliometric insights into gene therapy for rare inherited hematologic pathologies: focus on sickle cell disease, hemophilia, and thalassemia

**DOI:** 10.1186/s13023-025-03957-0

**Published:** 2025-11-04

**Authors:** Moutaz W. Sweileh

**Affiliations:** 1 Department of Pathology, Faculty of Medicine and Allied Health Sciences, An-Najah National University, Nablus, Palestine; 2 Department of Pathology, An-Najah National University Hospital, Nablus, Palestine

**Keywords:** Gene therapy, Inherited hematologic disorders, Gene editing, CRSPR/Cas9, Bibliometric

## Abstract

**Background:**

Inherited hematologic disorders such as sickle cell disease (SCD), thalassemia, and hemophilia are rare but devastating conditions with high morbidity and mortality. Advances in gene therapy have opened curative prospects. The objective of the current study was to provide a comprehensive bibliometric and knowledge-mapping analysis of global research on gene therapy targeting SCD, thalassemia, and hemophilia.

**Methods:**

A Scopus-based bibliometric search was conducted for the period from 1986 to 2024. The search used title-specific queries for the target diseases and title-abstract queries for gene therapy technologies. Bibliometric indicators and network visualization maps, created by VOSviewer, were presented.

**Results:**

A total of 1399 articles were retrieved. Publication growth revealed two phases: a steady, but slow phase (1986–2018), and mature expansion phase (2019–2024), with an average annual growth rate of 28% over the last decade. The articles had a mean of 47.6 citations/article and an H-index of 126. Research was mainly distributed across medicine (39.5%), biochemistry/genetics/molecular biology (30.7%), immunology (8.1%), and pharmacology/toxicology (7.5%), reflecting the multidisciplinary nature of the field. The most prolific journal was *Blood* (8.6%), followed by *Hemophilia*, *Molecular Therapy*, *Human Gene Therapy*, and *Blood Advances*. The United Stated dominated the field (n = 863; 61.7%), followed by China and the United Kingdom, with the United States showing strong intra-country collaborations, while European countries demonstrated high international collaborations, often with the United States. Leading institutions included the Children’s Hospital of Philadelphia (7.5%) and University of Pennsylvania (5.9%). Co-authorship analysis revealed robust collaboration networks, with notable clusters around AAV-based hemophilia therapy and CRISPR-mediated gene correction for hemoglobinopathies. Keyword co-occurrence highlighted themes like AAV vectors, genome editing, CRISPR-Case9, and ex vivo hematopoietic stem cell modification. Overlay visualization maps indicated a recent surge in CRISPR and clinical applications research.

**Conclusions:**

The bibliometric findings underscore the rapid evolution of gene therapy research for SCD, thalassemia, and hemophilia, moving from experimental approaches to clinical translation. The strong interdisciplinary collaboration, rising clinical trials, and emergence of genome editing tools suggest that the field is entering a transformative era, offering real-world therapeutic solutions for previously incurable inherited blood disorders.

## Background

Inherited hematologic disorders such as sickle cell disease (SCD), thalassemia, and hemophilia are rare diseases characterized by debilitating symptoms, lifelong morbidity, and, in some cases premature mortality [1, 2]. Globally, SCD is affecting people mainly in the Sub-Sharan Africa and the United States, especially in the African American communities [3]. Current treatment of the SCD include blood transfusion and hydroxyurea which are not curative. Thalassemia, is another hemoglobinopathy, caused by a mutation that reduce or eliminate the production of alpha or beta globin chains [4]. In beta-thalassemia, patients require lifelong blood transfusion and iron chelation. The disease is primarily present in people living in the Mediterranean area.

Hemophilia is an X-linked bleeding disorder caused by deficiencies in clotting factors VIII (hemophilia A) or IX (hemophilia B) [5]. The disease affects mainly males and leads to spontaneous bleeding and joint damage. Standard therapy includes frequent administration of missing clotting factors. These three diseases arise from well-defined single gene mutation, making them ideal candidates for gene therapy, a field that seeks to correct, replace, or compensate, for defective genetic material at the molecular level [6]. Gene therapy in the context of hematologic diseases is built upon a profound understanding of the molecular pathology of each condition. In SCD, a single nucleotide substitution (Glu6Val) in the Beta globin gene results in the production of abnormal hemoglobin S (HbS), leading to erythrocyte sickling, vaso-occlusion, and multi-organ damage [7]. Thalassemia, including alpha and beta-thalassemia arises from mutations that impair the synthesis of globin chains, leading to ineffective erythropoiesis and anemia [8]. Hemophilia A and B, on the other hand, are caused by mutations in the F8 and F9 genes respectively, resulting in deficient clotting factor VIII or IX activity and a tendency for spontaneous bleeding [9].

The principle of gene therapy for these disorders is either (1) to repair the defective gene (gene editing), or (2) replace it with a functional copy (gene addition) by integrating the functional copy into the target cell genome randomly, semi-randomly, or targeted, but the affected gene is not replaced, or (3) modulate gene expression to restore physiological function. Molecular approaches have evolved from early viral vector-mediated gene transfer to sophisticated genome editing technologies, which allow precise correction of pathogenic mutations in endogenous loci [6]. For SCD and thalassemia, a major therapeutic strategy involves the reactivation of fetal hemoglobin (FHb) through targeted disruption of regulators such as BCL11A, thereby bypassing the defective adult B-globin gene [10]. Alternatively, direct gene addition using lentiviral vectors encoding functional B-globin has reached clinical translation exemplified by betibeglogene autotemcel [11]. In the context of hemophilia, adeno-associated virus (AAV)—mediated gene therapy has achieved landmark success with therapies such as etranacogene dezaparvovec and valctocogene roxaparvovec delivering durable expression of factor IX and VIII, respectively and significantly reducing bleeding episodes [12, 13].

At the molecular level, successful gene therapy must address several challenges including efficient gene delivery to target cells, primarily haemopoietic stem cells for SCD and thalassemia, and hepatocytes for hemophilia), precise regulation of transgene expression to physiological levels, minimization of insertional mutagenesis risks, immune responses to viral vectors, and long-term durability of therapeutic effects [14, 15]. Recently, the introduction of gene editing techniques has opened new horizons for people with inherited genetic diseases including SCD, hemophilia, and thalassemia [16–20]. Genetic editing, also referred to as genome editing, is a transformative field of molecular biology that enables scientists to precisely alter an organism’s DNA. Through these techniques, specific segments of genetic material can be deleted, inserted, or replaced within the genome [21–24]. The journey of genetic editing began in the 1970 s with the advent of recombinant DNA technology, allowing for the first manipulation of genetic sciences using restriction enzymes. In the late twentieth century, more sophisticated tools emerged, including Zinc Finger Nucleases (ZFNs) and transcription activator-like effector nucleases (TALEN), both of which rely on engineered proteins to target and cleave DNA at specific locations [25]. However, these tools required complex protein engineering, limiting their widespread adoption. The landscape shifted dramatically in 2012 when Jennifer Doudna and Emmanuelle Charpentier introduced CRISPR-Cas9, a technology derived from the bacterial immune system that uses a guide RNA to direct nuclease to a specific DNA sequence, enabling highly targeted genome editing [21, 26–29].

Knowledge mapping and bibliometric analysis are two complementary methodologies used to systematically evaluate the landscape of scientific research on a specific topic. Knowledge mapping refers to the process of visualizing structure, development, key thematic areas within a body of literature, helping researchers identify how knowledge is generated, interconnected, and evolving over time. Meanwhile, bibliometric analysis quantitatively assesses publications using metrics such as average annual growth rate (AAGR), citation counts, and key contributors [30]. Unlike traditional narrative reviews, which are often subjective and selective, or systematic reviews, which focus narrowly on clinical outcomes or interventions using strict inclusion criteria, bibliometric analysis provides a broad, objective, and data-driven overview of the structure and dynamics of scientific research [31, 32]. Bibliometric analysis is particularly useful in fast evolving fields such as gene therapy in rare diseases, where the volume and diversity are rapidly expanding. The objective of the current study is to provide a comprehensive insight of the current research landscape on gene therapy in relation to SCD, thalassemia, and hemophilia, identify key contributors and collaborative networks, and highlights emerging areas in the field. By doing so, the paper aims to guide future research priorities, support strategic funding decisions, and foster interdisciplinary collaboration. Moreover, the insights derived from this study have the potential to accelerate the development of bench-to-bedside translation, innovative diagnostic tools and therapeutic intervention of rare genetic diseases, many of which remain under-researched and under-served in global health agenda.

## Methodology

The current study adheres to the preliminary guideline for reporting bibliometric reviews of biomedical literature (BIBLIO) [33]. This guideline emphasizes methodological transparency, reproducibility, and scientific rigor by outlining essential components such as clear research objectives, defined search strategies, data source selection, and the use of validated bibliometric indicators. By adhering to BIBLIO, the study ensures its findings are both credible and aligned with emerging standards in bibliometric research.

### Study design

This study employed a bibliometric and knowledge-mapping design to systematically analyze global research trends related to gene therapy in SCD, thalassemia, and hemophilia. The approach integrates quantitative bibliometric indicators with network visualization techniques to provide a comprehensive overview of the scientific landscape. The focus was on identifying research output, collaboration patterns, thematic evolution, and citation impact from 1986 to 2024.

### Data source

The Scopus database (Elsevier) was selected as the sole source of data. Scopus offers broad coverage of biomedical and clinical research, detailed citation information, and advanced search capabilities that enable complex bibliometric retrieval. Its extensive indexing of peer-reviewed journals made it ideal for capturing relevant literature in the fields of molecular medicine, hematology, and gene therapy.

### Search strategy and keywords

A carefully structured search strategy was implemented to ensure the retrieval of focused and high-quality literature. The search combined terms related to target diseases (e.g., “sickle cell disease”, “sickle cell an*emia”, “thalassemia”, “h*emophilia”, “HbS”, “SCD”, “hemoglobinopath*”, “B-subunit”, “B-globin”,"factor VIII deficiency","factor IX deficiency","F8 gene","F9 gene", “Hb mutation”) and “gene therapy”–related terms (e.g. “genome editing”, “gene transfer”, “gene correct*”, “genetic editing”, Clustered Regularly Interspaced Short Palindromic Repeats","CRISPR Associated Endonuclease Cas9","CRISPR/Cas9", “CRISPR”,"TALENs","zinc finger nucleases","ZFNs","base editing","prime editing","nucleotide* editing", “DNA editing”,"viral vector","lentiviral vector","AAV vector","adeno-associated virus","hematopoietic stem cell gene therapy","ex vivo gene therapy","in vivo gene therapy"). The search for the target disorders utilized “TITLE” search strategy with certain restrictions while the search for the “gene therapy” utilized the “TITLE-ABSTRACT” search strategy. The full search query is available from the author upon personal request.

### Inclusion and exclusion criteria

Inclusion criteria required articles to be full-length original research articles, published in peer-reviewed journals, addressing gene therapy explicitly applied to SCD, thalassemia, or hemophilia. Exclusion criteria included non-English publications. The following types of documents were also excluded: notes, editorials, conference abstracts, books, book chapters, letter to the editor, review articles and non-journal document types.

### Validation and quality check

To ensure the validity and accuracy of the retrieved dataset, a rigorous validation and quality check process was undertaken. First, the search strategy was refined by using title-specific queries for the target diseases, which minimized the inclusion of irrelevant articles and thus reduced false-positive results. Conversely, for the broader term “gene therapy”, the search was conducted within the title-abstract field to capture relevant studies that may not explicitly mention “gene therapy” in the title field, thereby minimizing false negatives. To validate the search results both prolific journals and prolific authors were checked for their alignment with the field, and found to be relevant to the topic being investigated. Moreover, a review of the top most cited articles revealed that all were directly related to gene therapy advancement in SCD, thalassemia or hemophilia providing strong evidence of the accuracy and validity of the search strategy. These measures collectively ensured that the final dataset accurately represented the research landscape and minimized methodological biases, reinforcing the credibility and comprehensiveness of the current bibliometric analysis.

### Data extraction

Bibliometric metadata were exported from Scopus, including information on article titles, abstracts, authors, affiliations, publication years, journal sources, keywords, total citation counts, and funding acknowledgments. The resulting dataset formed the foundation for both descriptive bibliometric analysis and network visualizations.

### Bibliometric indicators

The following indicators were analyzed: annual trends in publication volume, leading countries, institutions, and authors contributing to the field, the most Influential journals based on volume and citation impact, citation analysis, including international collaboration patterns, identification of landmark publications and highly cited studies. Citation metrics, such as mean, median, range, and H-index, give insight into the influence and visibility of publications in the field. The H-index is defined as the highest number of publications (e.g. H) that have received at least H number of citations [34].

### Bibliometric visualization and network mapping

Visual bibliometric analyses were performed using VOSviewer (version 1.6.20) [35, 36]. Four main types of network maps were constructed and interpreted:

#### Co-authorship map

Co-authorship network map at the level of authors was generated for authors with a minimum contribution of five publications. The map was meant to visualize collaboration intensity and structure. Nodes represented authors or institutions, with node size proportional to the number of publications. Densely clustered nodes identified collaborative hubs, suggesting strong institutional or international research partnerships.

#### Author keyword co-occurrence map

Keyword co-occurrence map was generated for terms with a minimum occurrence of at least five times to reveal the conceptual structure of the field. Keywords were analyzed based on their frequency of appearance and their association with one another. The resulting thematic clusters highlighted dominant research area, such as CRISPR-mediated correction of beta-globin gene mutations, stem cell-based delivery strategies, and novel editing technologies like base or prime editing.

#### Co-occurrence maps of terms in titles/abstracts

This type of map visualizes the intellectual structure of a research field by identifying frequently occurring terms and their relationships. Clusters of related terms are color-coded revealing measure research themes or topics within the dataset. This method offers a powerful way to explore emerging trends, research focus areas, and the conceptual organization of the scientific literature. In the current study, the top 500 frequent terms in titles/abstracts of the retrieved articles were mapped.

#### Overlay visualization (temporal map)

Overlay visualization maps were created to display the temporal dynamics of keyword emergence and evolution. Earlier research themes appeared in cooler colors (blue/green), while more recent themes appeared in warmer colors (yellow/orange)/This allowed identification of emerging trends and shifts in research priorities over time.

Thresholds were applied during visualization to ensure clarity (e.g., a minimum number of documents per author, institution, or keyword), and normalization methods were used to balance differences in node weight and link strength across maps.

#### Ethical considerations

This study exclusively utilized bibliometric data derived from a publicly available and licensed database (Scopus). No human subjects, personal data, clinical records, or confidential information were accessed. Therefore, institutional review board approval was not required. All bibliometric data were analyzed and reported in aggregate form, without reference to individual researchers beyond standard bibliometric metrics (e.g., citation counts). The research process adhered strictly to established guidelines for bibliometric studies, ensuring scientific integrity, transparency, and reproducibility.

## Results

### Flow chart

**Fig. 1:**
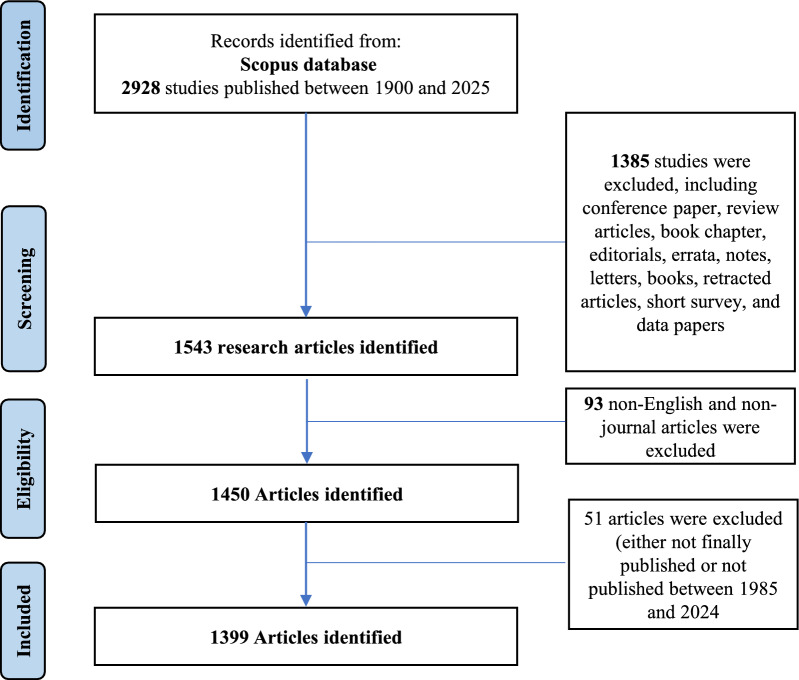
Flow diagram of the number of articles retrieved during the search strategy

Based on the search methodology, 1399 research articles were published between 1986 and 2024. Figure 1 is the flow diagram with the corresponding number of articles retrieved in each step in the search strategy.

### Disease-specific publications

Out of the 1399 publications included in the analysis, 714 (51.0%) addressed research topics related to hemophilia, while 674 (48.2%) publications addressed other disorders (SCD and thalassemia). Therefore, the retrieved publications showed balanced distribution of scientific research output between these two major areas of hematologic research. Of the 1399 retrieved articles, 762 (54.6%) were carried out in animals or cell lines representing pre-clinical research, while the remaining articles were carried out using human subjects, representing clinical research.

### Growth of publications and citations

**Fig. 2:**
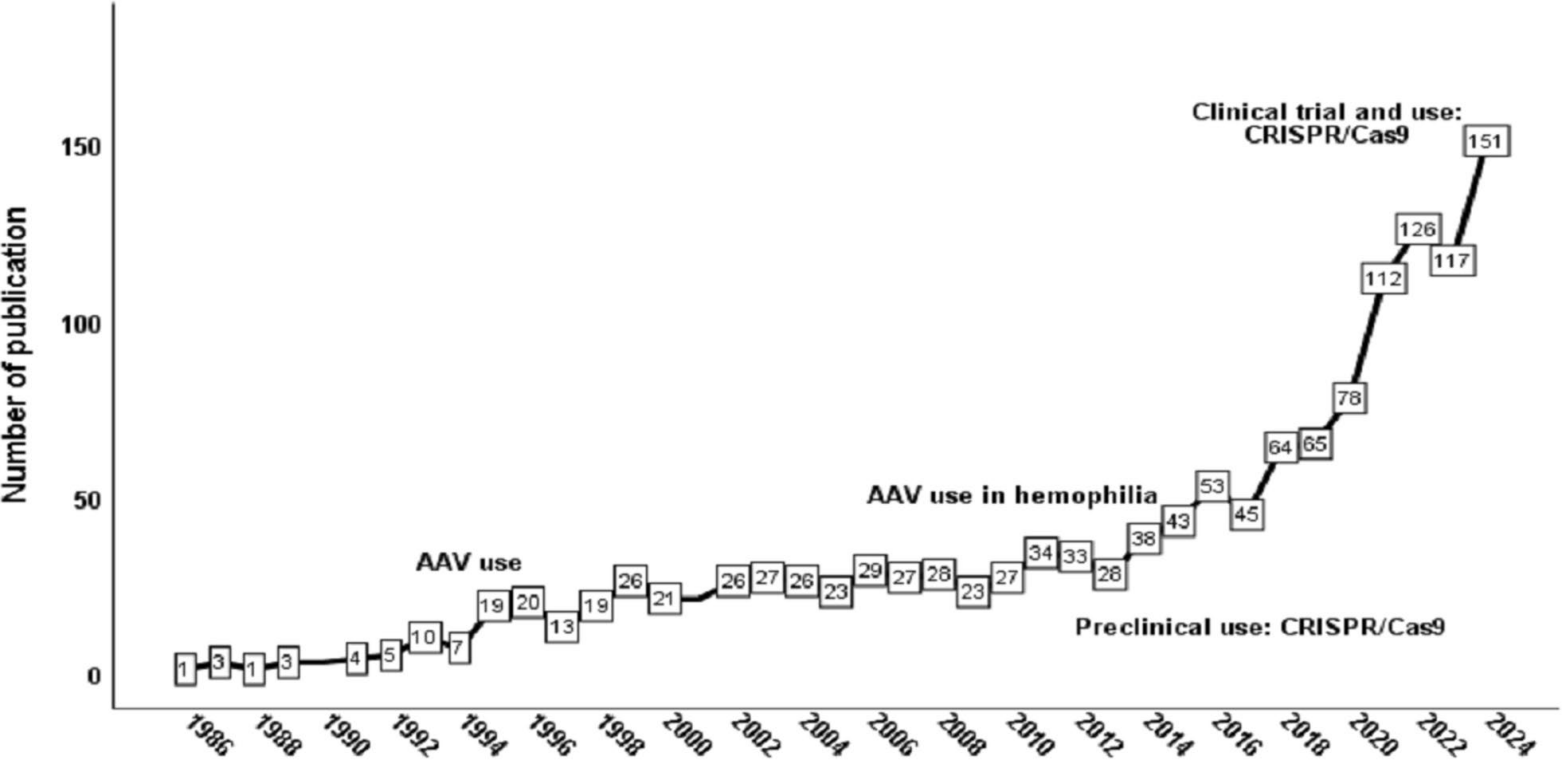
Annual growth of publications on gene therapy of inherited hematologic disorders

The annual growth of publications on gene therapy for inherited hematologic disorders, specifically SCD, thalassemia, and hemophilia, from 1986 to 2024, can be divided into two distinct phases based on the number of publications and the evolution of the field (Fig. 2). The first phase (1986–2018), represents steady, but slow increase in the number of publications. The second phase, from 2019 to 2024, is a mature expansion phase, characterized by a sharp surge in the number of publications. The AAGR in the last decade (2015–2024) was approximately 28.0%. The 1399 retrieved articles collected 66,538 citations with a mean of 47.6 citations per article and an H-index of 126.

### Subject areas

The distribution of the 1399 retrieved articles by subject area reveals the interdisciplinary nature of research on gene therapy for inherited blood disorders (Table 1). Medicine dominates the field, accounting for 39.5% of the total publications. The focus on medicine reflects the active translation of gene therapy approaches from bench to bedside, through clinical trials, treatment optimization, and regulatory pathways. Following closely, Biochemistry/Genetics/Molecular Biology represents 30.7% of the publications, showcasing the critical role of fundamental biological sciences in the development of gene therapy technologies. Immunology/Microbiology, comprising 8.1%, highlights the importance of immune response studies, particularly in understanding host-vector interactions, immune tolerance, and complications such as immune-mediated clearance of transduced cells. Pharmacology/Toxicology/Pharmaceutics accounts for 7.5% of the articles, reflecting research focused on the safety, biodistribution, dosing strategies, and toxicological profiles of gene therapy products. Regulatory approval processes for gene therapies heavily rely on robust pharmacological and toxicological data, making this a key area of research.

**Table 1:** Top subject areas of the retrieved articles

Rank	Subject area	Number of publications	% (N = 1399)
1st	Medicine	849	60.7
2nd	Biochemistry, Genetics and Molecular Biology	659	47.1
3rd	Immunology and Microbiology	173	12.4
4th	Pharmacology, Toxicology and Pharmaceutics	162	11.6
5th	Multidisciplinary	79	5.6

### Top cited articles

The analysis of the most cited articles showed that a dominant group of seminal works focused on gene therapy for hemophilia, particularly using AAV vectors to deliver functional copies of coagulation factor gene. The landmark study by Manno et al. [37] published in Nature Medicine stands out with 1839 citations pioneering the field by demonstrating the successful transduction of the human liver with an AAV vector expressing factor IX (FIX) in patients with severe hemophilia B. Building on this, Nathwani et al. [38, 39] used a self-complementary AAV vector to achieve more durable and clinically meaningful factor IX levels, significantly reducing bleeding rates and factor usage. Kay et al. [40] and Manno et al. [41] validated intramuscular AAV delivery. More recently George et al. [42] achieved sustained high-level expression using a high specific activity factor IX variant, while Rangarajan et al. [43] extended AAV gene transfer success to hemophilia A, overcoming the technical challenges related to large factor VIII gene. In parallel, the field of gene editing for hemoglobinopathies has rapidly advanced. Frangoul et al. [17] reported CRISPR-Cas9-based editing of the BCL11A enhancer, restoring fetal hemoglobin production and achieving transfusion independence and elimination of vaso-occlusive crises. Earlier, Cavazzana-Calvo [44] demonstrated lentiviral B-globin gene addition leading to long term transfusion independence in B-thalassemia. Dever et al. [45], provided essential preclinical evidence of CRISPR-mediated correction of HBB mutations in hematopoietic stem cells. Table 2 shows the top 10 cited articles and their citation metrics.

**Table 2:** Top 10 cited articles and their citation metrics

Title	Year	Source title	Total number of citations	Number of citations per year
Successful transduction of liver in hemophilia by AAV-Factor IX and limitations imposed by the host immune response	2006	Nature Medicine	1839	96.8
Adenovirus-associated virus vector-mediated gene transfer in hemophilia B	2011	New England Journal of Medicine	1578	112.7
CRISPR-Cas9 gene editing for sickle cell disease and β-thalassemia	2021	New England Journal of Medicine	1205	301.3
Transfusion independence and HMGA2 activation after gene therapy of human β-thalassaemia	2010	Nature	1161	77.4
Long-term safety and efficacy of factor IX gene therapy in hemophilia B	2014	New England Journal of Medicine	1097	99.7
Evidence for gene transfer and expression of factor IX in haemophilia B patients treated with an AAV vector	2000	Nature Genetics	906	36.2
CRISPR/Cas9 β-globin gene targeting in human haematopoietic stem cells	2016	Nature	711	79.0
AAV-mediated factor IX gene transfer to skeletal muscle in patients with severe hemophilia B	2003	Blood	635	28.9
Hemophilia B gene therapy with a high-specific-activity factor IX variant	2017	New England Journal of Medicine	599	74.9
AAV5-factor VIII gene transfer in severe hemophilia a	2017	New England Journal of Medicine	582	72.8

### Key contributors

The 1399 retrieved articles were disseminated by 375 scientific journals. However, more than one quarter (n = 360; 26.4%) of the retrieved articles were published by five specialized journals **(**Table 3**).** The top five prolific journals include three journals with clinical focus and two journals with pre-clinical focus. *Blood* emerged as the most prolific journal with 121 (8.6%) articles. The leading role of *Blood* journal (Impact Factor = 21.1) is expected given that it is an international forum for publication in basic, clinical, and translational hematology. Following closely, *Hemophilia* (Impact Factor = 3) contributed 75 (3.4%) articles. *Hemophilia* is a specialized journal in inherited bleeding disorders. The presence of this specialized journal in the top prolific list indicates the emphasis of scientific community on using gene therapy for the treatment of hemophilia A and B. *Molecular Therapy* (Impact Factor = 12.1) ranked third and contributed 68 (4.9%) articles. The journal focuses on cellular and gene therapy. Both *Human Gene Therapy* (Impact Factor = 4.1) and *Blood Advances* (the American Society of Hematology; Impact Factor = 7.4) ranked 4th and 5th respectively.

**Table 3:** Top five prolific journals

Rank	Journal name	Number of publications (%)	Main focus*
1st	*Blood*	121 (8.6)	Clinical aspects
2nd	*Haemophilia*	75 (5.4)	Clinical aspects
3rd	*Molecular Therapy*	68 (4.9)	Pre-Clinical Aspects
4th	*Human Gene Therapy*	64 (4.6)	Pre-Clinical Therapy
5th	*Blood Advances*	41 (2.9)	Clinical

^*^The main focus of the journal, clinical versus pre-clinical, was obtained from the journal homepage

At the country level, the United States showed a clear dominance in the field with 863 (61.7%) articles (Table 4). This overwhelming share by the United States emphasizes the presence of strong innovative research environment and well-built infrastructure for clinical trial investigation and regulatory policies. In addition, the strong emphasis on intra-country collaboration within the United States indicates the presence of many leading scientific research centers in the field. China follows distantly with 157 (11.2%) articles. China showed similar emphasis on intra-country collaboration. The remaining countries in the list (the United Kingdom, Italy, Canada, and France) showed high dependence on international collaboration in their contribution to the field.

**Table 4:** Top five prolific countries and extent of international research collaboration

Rank	Country	Number of publications (%)	Number of publications with intra-country collaboration (%)	Number of publications with inter-country (international) collaboration (%)
1st	United States	863 (61.7)	535 (62.0)	328 (38.0)
2nd	China	157 (11.2)	96 (61.1)	61 (38.9)
3rd	United Kingdom	147 (10.5)	44 (29.9)	103 (70.1)
4th	Italy	126 (9.0)	47 (37.3)	79 (62.7)
5th	Canada	89 (6.4)	16 (18.0)	73 (82.0)
5th	France	89 (6.4)	20 (22.5)	69 (77.5)

A total of 11,338 author names were recorded across the 1399 articles, with a mean number of articles of 8.1 authors per article, indicating a high level of collaborative research activity. The majority of publications (n = 953, 68.1%) involved five or more authors per article, underscoring the multidisciplinary and collaborative nature of research in this field. In terms of individual researchers, K. A. High is the leading figure with 39 publications [46, 47]. Her work has been pivotal in the development and clinical translation of AAV-based gene therapy in haemophilia. T.C. Nicolas follows with 36 publications. His research focused on large animal model of gene therapy on bleeding disorders [48, 49]. J.F. Tisdale ranked third with 30 (2.1) publications that focused mainly on hematopoietic stem cell transplantation and gene editing approaches for hemoglobinopathies such as SCD and thalassemia [50, 51].

Table 5 shows the list of top prolific institutions in the field. The list shows that research output is focused on a small group of academic and clinical centers based in the United States. The Children’s Hospital of Philadelphia (CHOP) (n = 105; 7.5%) ranked first. The University of Pennsylvania ranked second (n = 83; 5.9%), emphasizing the leading role of academic and clinical institutions in Pennsylvania in gene therapy in inherited genetic blood disorders. These institutions stand out as the powerhouse behind many of the efforts in AAV-mediated gene therapy for hemophilia, and it is closely associated with outstanding researchers including Katherine A. High and Valder R. Arruda the most prolific authors identified in the analysis. The close partnership between CHOP and the University of Pennsylvania, particularly through the Perelman school of Medicine, has created a highly synergistic environment, accelerating advances in vector design, clinical trials, and regulatory milestones.

**Table 5:** Top five prolific institutions in the field

Rank	Institution	Number of publications (%)
1st	*The Children's Hospital of Philadelphia*	105 (7.5)
2nd	*University of Pennsylvania*	83 (5.9)
3rd	*The University of North Carolina at Chapel Hill*	75 (5.4)
4th	*University of Washington*	72 (5.1)
5th	*Harvard Medical School*	64 (4.6)

### Author collaboration network

**Fig. 3:**
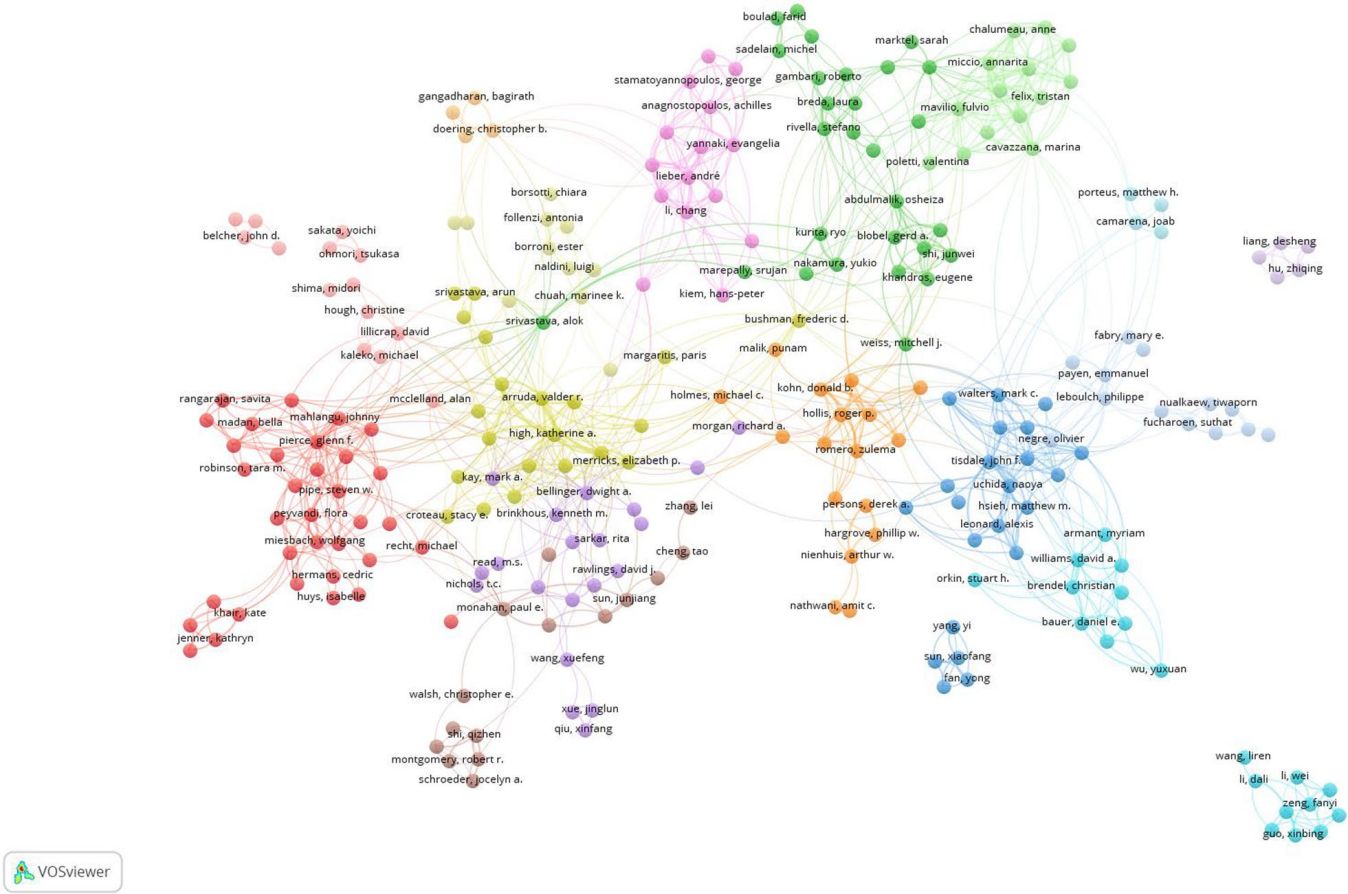
Network visualization map of author collaboration network. Authors with a minimum contribution of five articles were included in the map

Figure 3 is a network visualization map of author co-authorship among researchers with a minimum contribution of five publications in the field. The map has 257 researchers distributed across 16 clusters. Each cluster consists of researchers who co-publish together. The red cluster is the largest one with 32 researchers. The cluster includes Glenn F. Pierce, Flora Peyvandi, Wolfgang Meisbach, and Steven W. Pipe. The cluster includes researchers from North America, Europe, and Australia. Therefore, the red cluster represents an international research collaborative network. This cluster focuses on gene therapy related to hemophilia. The cluster is clinically driven with less focus on molecular mechanism or vector design. The yellow cluster is one of the most central and influential in the field give the large nodes present in the cluster. The cluster includes Katherine A. High, Valder R. Arruda, and Timothy C. Nicholas. Researchers in the yellow cluster and closer clusters were involved in both basic and clinical research in gene therapy in hemophilia and other blood disorders. The green cluster includes several researchers with distinct research activity, such as Michel Sadelin, Guiliana Ferrari, Annarita Miccio, Fulvio Mavilio, Stefano Rivella, and Marina Cavazzana. Researchers in this cluster are known for their research activity in the development of lentiviral vector development, gene transfer into hematopoietic stem cells, and clinical application in hemoglobinopathies. This cluster represents researchers from North America and Europe. The orange cluster is led by Donald B. Kohn, Roger P. Hollis, Derek A. Persons, Arthur W. Neinhuis, and others. This cluster of researchers focused on lentiviral gene addition for treatment of hemoglobinopathies.

### Author keywords co-occurrence

**Fig. 4:**
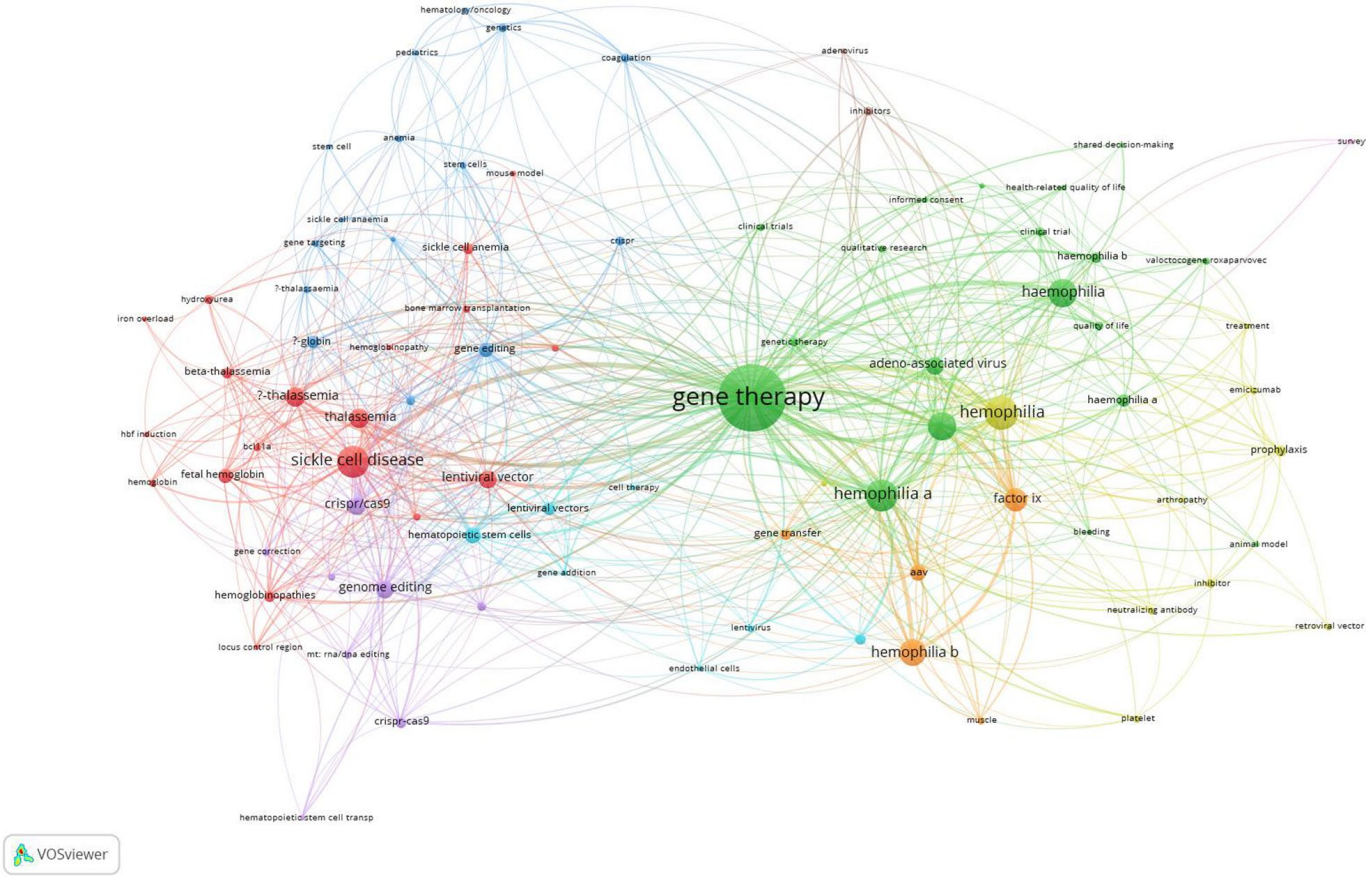
Network visualization map of author keywords co-occurrences. Keywords with a minimum occurrence of five were included. The map includes 86 keywords

Figure 4 is author keywords co-occurrence map of the retrieved articles on gene therapy of inherited blood disorders. The map included author keywords with a minimum occurrence of five (n = 86). Large nodes in the map refers to the main research hotspots in the retrieved articles. At the center of the map, the term “gene therapy” emerges as the most prominent and highly interconnected keyword. To the right of the map, keywords such hemophilia. hemophilia A, hemophilia B, factor IX, and adeno-associated virus. The right side of the map also includes keywords such as clinical trial, quality of life, and treatment. These keywords together refers to AAV-mediated gene therapy in hemophilia and its clinical impact in humans. On the left side of the map, SCD, thalassemia, CRISPR/Cas9, hematopoietic stem cells, lentiviral vector, and genome editing form the core of the therapeutic strategy of inherited blood disorder involving ex vivo genetic modification of stem cells using CRISPR/Cas9 base editing or lentiviral gene addition. The left side of the map also includes other keywords such as fetal hemoglobin, HbF induction, and bone marrow transplantation reflecting the therapeutic strategy focusing on fetal hemoglobin reactivation and stem cell manipulation. The overlay visualization map of the keywords co-occurrence indicates that both gene editing, clinical trial, and CRISPR/Cas9 were the most recently (yellow colors) introduced keywords.

### Title and Abstract Terms Co-occurrence

**Fig. 5:**
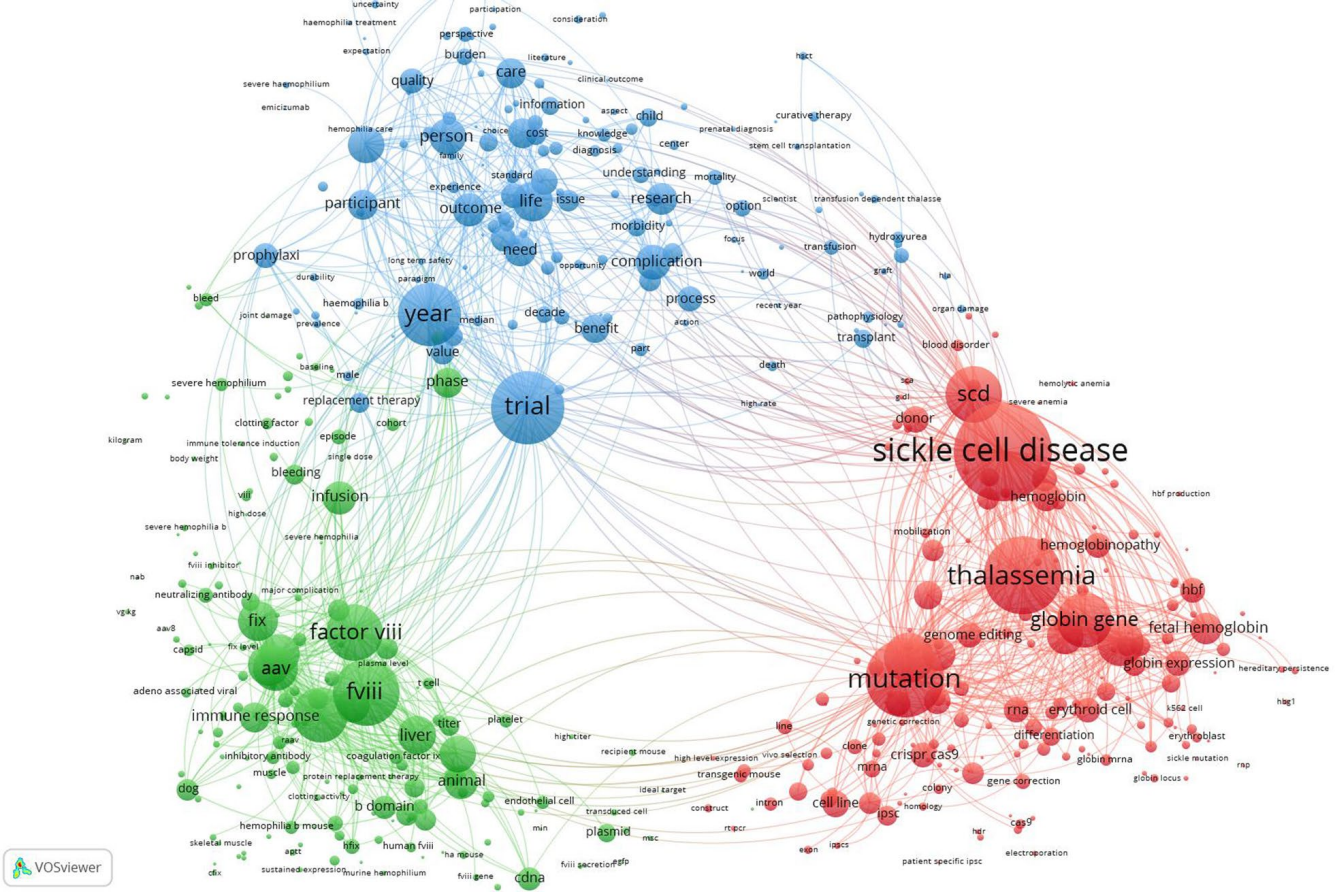
Network visualization map of terms in titles and abstracts of the retrieved articles. The map includes 500 frequent terms

Figure 5 is a network visualization map of frequent terms in titles and abstracts of the retrieved articles. The map has three distinct clusters with different colors. The red cluster, the right side of the map, represents research cantered on SCD and thalassemia with key terms such as “sickle cell disease”, thalassemia, mutation, hemoglobin, “genome editing”, CRISPR/Cas9, and “fetal hemoglobin”. The green cluster on the left side of the map is centered around hemophilia with the following keywords “hemophilia A”, “hemophilia B”, “factor VIII”, “factor IX”, “infusion”, “AAV”, “immune response”, and “liver”. Other keywords such as safety and neutralizing antibodies, and vector design point to the challenges in the field of gene therapy. The blue cluster is located centrally between the red and blue clusters. The cluster includes terms such as trial, outcome, life person, management, quality, care, and complication. This research cluster captures the transition of gene therapy from experimental stages to clinical application, with emphasis on clinical trials, patient-centered outcomes, management of complications, and quality of life improvements.

## Discussion

This bibliometric analysis was conducted to systematically map and evaluate the research landscape of gene therapy for inherited hematologic disorders, with a focused lens on SCD, thalassemia, and hemophilia. By analyzing publication trends, citation patterns, key contributors, and thematic developments, the study aims to uncover the driving force, collaborative networks, and emerging innovations that have shaped this dynamic field. The findings not only reflect the maturation of gene therapy research, but also highlight the profound shift from the technological challenges to recent clinical breakthroughs, underscoring the informative potential of gene editing and advanced vector systems in addressing these rare life-threatening genetic diseases.

The growth of publications in the field was characterized by slow growth before 2017 which can be attributed to several key and technical challenges that limited early progress [52]. During the initial decades of gene therapy research, significant barriers existed, including inefficient gene delivery systems, limited control over transgene expression, immune reactions to vectors, and safety concerns related to insertional mutagenesis [53]. Early gene therapy efforts primarily relied on retroviral vectors, which, while capable of stable integration into host genomes, carried high risks of oncogenesis, as tragically evidenced in early clinical trials where patients developed leukemia after gene therapy of immunodeficiencies [54–56]. These setbacks led to increased regulatory caution, heightened public skepticism, and general slowdown in clinical development across the field. Another important factor was the complexity of hematologic disorders themselves. Diseases such as SCD and thalassemia involve intricate regulation of globin gene expression, necessitating not just gene addition but fine-tuned control of transcriptional environments in hematopoietic stem cells [17, 57]. Similarly, hemophilia gene therapy efforts were constrained by challenges in achieving therapeutic levels of clotting factors without provoking neutralizing immune responses to the introduced proteins or viral vectors [58, 59]. Additionally, the technology to isolate, manipulate, and safely reintroduce hematopoietic stem cell ex vivo was still in its infancy during the 1990 s and early 2000 s [60–63]. A key distinction in gene therapy strategies for rare hematological genetic diseases lies in the mode of delivery and cellular targets. Hemoglobinopathies such as ß-thalassemia and SCD primarily rely on ex vivo gene therapy approaches, where autologous hematopoietic stem and progenitor cells (HSPCs) are harvested from the patient, genetically modified outside the body using lentiviral vectors or CRISPR/Cas9-based genome editing tools, and then reinfused following myeloablative conditioning. This approach allows for precise editing or insertion of therapeutic transgenes such as anti-sickling ß-globin variants or reactivation of fetal hemoglobin through BCL11A disruption. However, ex vivo therapy is limited by challenges in stem cell mobilization, efficient transduction/editing, toxicity of conditioning regimens, and the need for specialized infrastructure for cell manipulation and transplantation [45, 64]. In contrast, gene therapy for hemophilia A and B has predominantly employed in vivo approaches, where AAV vectors are administered systemically to deliver functional copies of F8 or F9 genes directly to hepatocytes. This strategy eliminates the need for stem cell manipulation but introduces unique bottlenecks, notably pre-existing immunity to AAV capsids, vector dose limitations due to hepatotoxicity, and the risk of immune responses against the transgene product or vector components, which may attenuate therapeutic efficacy or necessitate immunosuppression. These differences underscore the disease-specific tailoring required in gene therapy design, and how the cellular targets (stem cells vs. hepatocytes) and delivery strategies (ex vivo vs. In vivo) critically shape the risks, challenges, and clinical implementation pathways [65–67].

The steep rise in the growth of publications after 2018 reflects a scientific turning point driven by major technological innovations and clinical successes. The development of AAV vectors, particularly serotype-optimized and liver-targeted variants, dramatically improved the safety and efficiency of gene transfer, making hemophilia gene therapy a tangible clinical reality [38, 43]. For SCD and thalassemia, the emergence of CRISPR-Cas9 genome editing enabled precise correction oh disease-causing mutations or reactivation of fetal hemoglobin pathways, providing curative strategies at the genetic level [17, 45, 68–74]. These advances were further supported by improved hematopoietic stem cell isolation, expansion, and transplantation techniques, which allowed for effective ex vivo editing followed by successful engraftment [17, 45, 68–74]. Importantly, regulatory pathways evolved to accommodate gene therapies more efficiently, culminating in the approval of multiple gene therapy products for inherited blood disorders, such as Zynteglo (betibeglogene autotemcel for B-thalassemia) and Hemgenix (etranacogene dezaparvovec for hemophilia B) [12, 75–79]. These approvals validated the feasibility and safety of gene therapy, spurring an influx of academic research, clinical trials, and industrial investment. Collectively, the rapid growth seen in the past few years reflects the convergence of safer vector system, powerful genome editing tools, improved clinical delivery methods, favorable regulatory shifts, and successful high-profile clinical trials, all of which reinvigorated global research interest and confidence in the promise of gene therapy for inherited hematologic disorders.

The dominance of subject areas such as medicine, biochemistry, genetics and molecular biology, immunology and microbiology, and pharmacology, toxicology, and pharmaceutics in the retrieved articles can be explained by the intrinsic multidisciplinary nature of the field and the biological complexity of these diseases. First and foremost, medicine was the leading subject area because gene therapy is fundamentally aimed at treating or curing clinical diseases. The clinical translation of gene therapy, from laboratory innovation to patient-centered interventions, ensures that medical journals remain the primary platform for disseminating findings [80–82]. Second, the strong presence of biochemistry, genetics, and molecular biology reflects the essential molecular underpinnings of gene therapy. These disorders arise from single-gene mutations (such as the B-globin gene mutation in SCD or mutations in F8/F9 in hemophilia), making the understanding of DNA structure, transcriptional regulation, portion function, and vector engineering critical for any therapeutic advancement [70, 83–86]. Fundamental research on viral vectors (e.g., AAV, lentivirus), gene editing technologies (e.g., CRISPR-Case9, base editing), and the molecular mechanisms of hemoglobin switching (e.g., BCL11A targeting) underlies most therapeutic strategies [70, 84–86]. Without this molecular groundwork, clinical gene therapy would not be feasible. Third, the involvement of immunology and microbiology stems from the immune challenges associated with gene therapy. Delivery vectors such as AAV and lentiviruses often trigger immune responses that can neutralize therapeutic effects or lead to adverse events [87–90]. The understanding and management of host immune responses, vector immunogenicity, and immune tolerance induction are therefore crucial components of successful and durable gene therapy [65, 91, 92]. Finally, the significant share of articles categorized under Pharmacology, Toxicology, and Pharmaceutics reflects the rigorous safety and dosing studies required for advancing gene therapies toward regulatory approval [93, 94]. Pharmacological studies are needed to assess biodistribution, optimal dosing regimens, vector persistence, and toxicity profiles, while toxicological assessments are vital for evaluating the risks of insertional mutagenesis, off-target effects of genome editing, and long-term adverse outcomes [95, 96].

The identification of leading journal such as *Blood*, *Hemophilia*, *Molecular Therapy*, *Human Gene Therapy*, and *Blood Advances* among the most prolific sources for publications on gene therapy for inherited blood disorders is not coincidental, but rather reflects the scientific maturity, clinical focus, and specialized audience these journals serve. *Blood* has broad readership that includes both basic scientists and clinicians, making it an ideal venue for studies that bridge laboratory discoveries and patient-centered applications. *Hemophilia’s* strong presence among the leading journals is explained by its niche specialization. As a journal specifically dedicated to bleeding disorders, it provides a focused platform for reporting advances in gene replacement therapy, factor IX and VIII gene delivery, and clinical outcomes in hemophilia patients. *Molecular Therapy*, the official journal of American Society of Gene and Cell Therapy (ASGCT), is another expected leader. it has established itself as a premier outlet for cutting-edge advances in gene transfer technologies, genome editing, and cellular therapies, As the field transitioned from experimental vectors to clinically validated therapies, *Molecular Therapy* became the natural choice for high-impact publications on vector optimization, CRISPR-based interventions, and first-in human gene therapy trials or both hemoglobinopathies and bleeding disorders. Finally, *Blood Advances* -an open-access journal launched by the American Society of Hematology-has rapidly gained attention due to its fast publication timelines and wide accessibility. Its emergence among the top journals reflects the growing demand for high-visibility, open = -access dissemination of gene therapy results, particularly as the field has entered a stage of clinical validation and patient-centered outcomes research.

The dominance of the United States in the field can be understood in the context of scientific leadership, national research priorities, funding mechanisms, clinical infrastructure, and international collaboration dynamics [97–100]. Historically, the United States has been the birthplace of many key technological innovations such as AAV vector development, lentiviral vector optimization, and early clinical trials in gene therapy. In addition, major institutions in the United States like the Children’s Hospital of Philadelphia, University of Pennsylvania, NIH, and Harvard Medical School have been heavily invested in translational research, offering robust infrastructures that combine cutting-edge laboratory science with world-class clinical trial capabilities. Furthermore, significant governmental (e.g., NIH) and private sector funding (e.g., through biotechnology firms) has sustained the field through the decades. In addition, regulatory flexibility, evident in the U.S. FDA’s evolving pathways for gene therapy approval, and strong industry-academic partnerships have accelerated both basic research and clinical translation, helping U.S.-based researchers maintain a self-sufficient, strong intra-country collaboration network.

The maps clearly revealed three dominant and interconnected thematic clusters. One cluster was centered on hemophilia gene therapy, reflecting the mature clinical development of AAV-based gene therapies for hemophilia, where therapeutic products have already reached late-phase trials and regulatory approval. The second major cluster was associated with SCD and thalassemia, demonstrating a newer and rapidly expanding frontier, where precision genome editing and ex vivo hematopoietic stem cell modification are offering curative prospects for hemoglobinopathies. The third cluster, more clinical in nature, signals the field’s growing maturity and a shift toward evaluating not just molecular correction but meaningful patient-centered outcomes. While hemophilia therapies have converged largely around AAV-mediated liver-directed delivery, hemoglobinopathy gene therapies are diverging into multiple strategies, gene addition, fetal hemoglobin reactivation, and direct gene correction, reflecting a highly innovative and competitive research landscape. This cluster indicates that gene therapy is no longer viewed as experimental but is being assessed for integration into routine clinical care, requiring new models of follow-up, ethics, and long-term patient support.

The overlay visualization maps added a temporal dimension to this understanding, showing that early research (pre-2015) was dominated by viral vector development, preclinical modeling, and basic gene transfer studies, while recent years (2018 onward) have witnessed the explosion of genome editing research, the translation of gene therapies into clinical practice, and the emergence of long-term outcome studies. The recent dominance of keywords like CRISPR/Cas9, gene editing, clinical trial, and quality of life indicates that the field has moved beyond technical feasibility to real-world application and longitudinal evaluation. The field has transitioned from a technology-focused phase (vector development, delivery efficiency) to a clinical impact phase (long-term outcomes, regulatory approval, and patient experience). For hemophilia, gene therapy has neared full clinical translation, with some therapies already commercialized. For SCD and thalassemia, the field is mid-transition, with CRISPR-based and lentiviral therapies moving rapidly from phase I/II studies into late-stage clinical evaluation.

The manuscript presents a strong and timely contribution to the field. One of the major strengths of the manuscript lies in its focused topic, which addresses a transformative area of research that is reshaping therapeutic approaches for rare, life-threatening disorders. The study provides a robust bibliometric analysis, offering a quantitative and evidence-based evaluation of the evolution of gene therapy over nearly four decades. By systematically analyzing the growth trends, subject areas, leading journals, authors, institutions, and collaboration networks, the manuscript moves beyond a descriptive narrative to deliver a structured and insightful mapping of the field’s development. Another point of strength is the clear thematic organization of the findings, supported by keyword and term co-occurrence maps that reveal the scientific landscape and major research clusters. Particularly valuable is the way the manuscript connects scientific progress with clinical translation, emphasizing the transition from early technological innovation to a mature phase where patient outcomes and quality of life considerations are increasingly prioritized.

### Limitation

While the current bibliometric analysis offered valuable insights in the field, certain limitations must be acknowledged. First, the study relied solely on one database, Scopus. Therefore, not all publications in the field might have been captured. Second, the language restrictions might have created language bias. Thirdly, the search strategy could have retrieved false-positive results and the possibility of false-negatives remain a possibility.

## Conclusion

The current bibliometric study systematically mapped the research landscape of gene therapy in inherited hematological disorders over the past four decades. The evolution showed a dynamic pattern with rapid expansion characterized by the introduction of novel technologies. The United States was the overwhelming leader with high intra-country collaboration. The contribution of countries in Europe and North America was dependent heavily on international research collaboration. Research output was heavily concentrated in a few specialized journals and was led by prominent institutions in the United States. A strong interdisciplinary nature of the field was evident as publications were distributed across different subject areas. Co-occurrence map showed that AAV-based gene therapy in hemophilia constituted a separate theme while ex vivo genome editing of hemoglobinopathies constituted the second major theme. Temporal analysis indicated that there is a time shift in research from early vector development in gene therapy into clinical applications and outcome. This analysis provides an essential resource for understanding the maturation of gene therapy research in inherited hematologic disorders and underscores emerging trends that will shape the future, including genome editing and its clinical translation in other rare diseases.

## Data Availability

Not applicable.

## Abbreviations

**SCD**: Sickle cell disease
**AAV**: Adeno-associated virus
